# Correlation of rosacea with hot beverage intake

**DOI:** 10.1002/ski2.154

**Published:** 2022-09-08

**Authors:** Ahmet Miguel Yildirim, Wei Fang, Michael S. Kolodney

**Affiliations:** ^1^ West Virginia University Health Sciences Center (Dermatology) Morgantown West Virginia USA; ^2^ West Virginia University West Virginia Clinical and Translational Science Institute Morgantown West Virginia USA

## Abstract

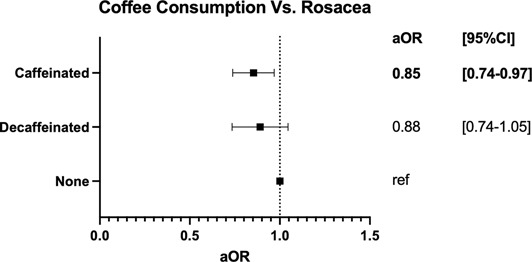

In this report, we correlated the incidence of rosacea with coffee (regular and decaffeinated) and tea consumption in a large cohort of middle‐aged men and women living within the United Kingdom. Caffeinated coffee drinkers had lower odds for rosacea diagnosis compared to non‐coffee drinkers. We hypothesize that the vasoconstrictive effects of caffeine in regular coffee overpower the vasodilatory effects associated with hot beverages and support it to be protective against rosacea.

## ETHICS STATEMENT

The UK Biobank was approved by the National Health Service (NHS) National Research Ethics Service. [Correction added on 27‐October‐2022, after first online publication: Ethics statement was added.]

To the Editor

Rosacea is a chronic inflammatory disease that is often associated with symmetrical facial erythema. Current data regarding the effects of coffee intake on rosacea are conflicting. Wilkin^1^ concluded that the active agent in coffee that causes flushing was heat rather than caffeine. Years later, Abram *et al*.^2^ found no significant relationship between caffeine intake and rosacea whereas Li *et al.* ^3^ found that caffeine from coffee was protective for rosacea. Moreover, decaffeinated coffee, caffeinated coffee and tea are rarely compared in the same study.^3^ In this report, we correlated the incidence of rosacea with coffee (regular and decaffeinated) and tea consumption in a large cohort of middle‐aged men and women living within the United Kingdom.

We analysed data from the UK Biobank^4^ (project #66911) where coffee status, tea status, and covariates were collected via a touchscreen questionnaire from 2006 to 2010. Rosacea diagnoses were determined from both inpatient and outpatient records in the period four years prior to four years after the participants completed the touchscreen questionnaire (2002–2014). After exclusion of participants who voluntarily withdrew and those with no information regarding coffee status and tea status, the final study population included 498,437 of the 502,427 original participants. Data was adjusted for age, sex, body mass index, ethnicity, smoking status, alcohol intake, and physical activity. Tea consumption and coffee consumption were utilised as covariates for one another in separate models. Non‐coffee drinkers and non‐tea drinkers were chosen as the reference groups for the effects of coffee and tea respectively.

**FIGURE 1:**
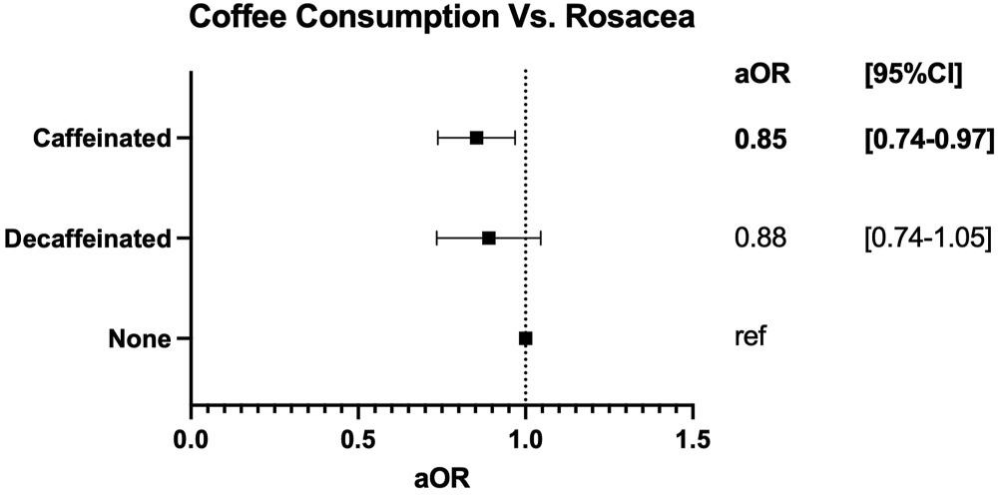
Forest Plot depicting the adjusted Odds Ratios (aORs) and 95% Confidence Intervals of caffeinated coffee drinkers and decaffeinated coffee drinkers compared to non‐coffee drinkers in UK Biobank from 2006 to 2010

As shown in Figure 1, caffeinated coffee drinkers had lower odds for rosacea diagnosis compared to non‐coffee drinkers (adjusted Odds Ratio (aOR) [95% Confidence Interval]: 0.85 [0.74–0.97]; *p* = 0.018). The difference in rosacea between decaffeinated and non‐coffee drinkers was not significant (aOR [95%CI]: 0.88 [0.74–1.05]; *p* = 0.145). No significant difference in rosacea diagnoses was seen when tea drinkers were compared to non‐tea drinkers (aOR [95%CI]: 1.01 [0.85–1.20]; *p* = 0.889) (data not shown).

The effect of caffeine intake on rosacea, whilst observing tea consumption, has been previously evaluated by Li *et al.* ^3^ In that study, caffeine intake from coffee was also inversely associated with the risk of rosacea in women. Caffeine is thought to promote vascular tone via antagonising adenosine receptors in the head and neck^5^ whereas heat is thought to vasodilate cutaneous blood vessels.^6^ As such, we hypothesise that the vasoconstrictive effects of caffeine in regular coffee overpower the vasodilatory effects associated with hot beverages and support it to be protective against rosacea. It is important to understand the relationship between caffeine intake and rosacea because many physicians possibly erroneously advise patients to avoid all hot beverages despite the potential benefit of coffee drinking. Moreover, caffeine may have potential as a novel treatment.

Our study suggests that caffeinated coffee consumption has an inverse association with rosacea. The strengths of this study include the large sample size and the comparison of rosacea with both coffee and tea intake habits. However, participation in the UK Biobank was voluntary so these self‐selected subjects may differ from the general population. Moreover, the self‐reported dietary habits, at the time of touchscreen questionnaire, may be different from those at the time of rosacea diagnosis. Future studies are needed to address the effects of caffeine on both the inflammatory and vascular symptoms of rosacea.

## CONFLICT OF INTEREST

The author declares that there is no conflict of interest that could be perceived as prejudicing the impartiality of the research reported.

## AUTHOR CONTRIBUTIONS

**Ahmet Miguel Yildirim**: Conceptualisation (Lead); Data curation (Lead); Investigation (Lead); Methodology (Lead); Writing – original draft (Lead). **Wei Fang**: Formal analysis (Lead). **Michael S. Kolodney**: Project administration (Lead); Supervision (Lead); Writing – original draft (Supporting); Writing – review & editing (Lead).

## FUNDING INFORMATION

William Weldon Endowment Fund.

## Data Availability

The data that support the findings of this study are available from UK Biobank. Restrictions apply to the availability of these data, which were used under license for this study. Data are available https://www.ukbiobank.ac.uk/ with the permission of the UK Biobank.
